# Effectiveness evaluation of quota payment for specific diseases under global budget: a typical provider payment system reform in rural China

**DOI:** 10.1186/s12913-018-3415-0

**Published:** 2018-08-13

**Authors:** Hao-miao Li, Ying-chun Chen, Hong-xia Gao, Yan Zhang, Liangkai Chen, Jing-jing Chang, Dai Su, Shi-han Lei, Di Jiang, Xiao-mei Hu

**Affiliations:** 1 School of Medicine and Health Management, Tongji Medical College, Huazhong University of Science and Technology, Wuhan, 430030 Hubei China; 2 Research center for Rural Health Services, Hubei Province Key Research Institute of Humanities and Social Sciences, Wuhan, 430030 Hubei China; 3 Department of Nutrition and Food Hygiene, Hubei Key Laboratory of Food Nutrition and Safety, and Ministry of Education Key Lab of Environment and Health, School of Public Health, Tongji Medical College, Huazhong University of Science and Technology, Wuhan, 430030 Hubei China

**Keywords:** Quota payment for specific diseases, Global budget, NCRMS, Provider payment system reform

## Abstract

**Background:**

Quota payment for specific diseases under global budget is one of the most typical modes of provider payment system reform in rural China. This study aimed to assess this reform mode from aspects of the total fee, structure of the fee and enrollees’ benefits.

**Methods:**

A total of 127,491 inpatient records from 2014 to 2016 were extracted from the New Rural Cooperative Medical Scheme (NRCMS) database in Weiyuan County, Gansu Province. Total fee, actual compensation ratio, out-of-pocket ratio, constituent ratio of the treatment fee, constituent ratio of the inspection and laboratory fee, and length of stay were selected as dependent variables. Both generalized additive models (GAMs) and multiple linear regression models were used to measure the change in dependent variables along with year.

**Results:**

Prior to the adjustment of the compensation type, out-of-pocket ratio and length of stay decreased, while total fee, actual compensation ratio, constituent ratio of the treatment fee, and constituent ratio of the inspection and laboratory fee increased. After the compensation type was adjusted, the mean of the total fee increased rapidly in 2015 and remained stable in 2016. The mean length of stay increased in 2015 but decreased in 2016. A comparison of inpatients suffering from diseases covered by quota payments and those suffering from general diseases revealed that total fee, out-of-pocket ratio, and length of stay decreased and actual compensation ratio increased for the former, whereas the opposite was true for the latter. Constituent ratio of the treatment fee and constituent ratio of the inspection and laboratory fee increased for both samples, except for the constituent ratio of the inspection and laboratory fee of quota payment diseases in 2016, which did not change.

**Conclusions:**

Quota payment for specific diseases under global budget had obviously positive effects on cost control in Weiyuan, Gansu. Considering the limited coverage of quota payment for diseases, the long-term effect of this reform mode and its replicability awaits further evaluation.

## Background

Over the past 20 years, China’s basic medical insurance systems, which consists of Urban Employees’ Basic Medical Insurance (UEBMI), Urban Residents’ Basic Medical Insurance (URBMI) and the New Rural Cooperative Medical System (NRCMS), have made remarkable achievements and have been highly affirmed by the whole world [1]. By the end of 2014, the three basic medical insurance systems had covered a population of 1.334 billion, which accounted for 96.3% of the population of China [2]. In particular, the NRCMS, established in 2002, has come up with numerous flagship policies aimed at rural populations [3, 4]. It expanded rapidly, with coverage increasing from 9.5% in 2003 to 98.9% of rural residents in 2013 [5]. Under NRCMS, rural residents’ access to health services has been facilitated significantly, while financial burden associated with seeking care were alleviated and equity in utilization of health services improved. Hence, health status of rural residents has been significantly improved [6–8].

Undoubtedly, the construction of NRCMS is one of the most important moves in China’s healthcare system reform. Moreover, the provider payment system plays an essential role in achieving the effectiveness of NRCMS, [9] as it can strongly affect the behavior of physicians [10]. Many studies have been conducted by the health management department at all levels, and the following are main reform trends. (1) Transfer from a post payment system to a prospective payment system. A post payment system, which was previously widely implemented in rural China, seemed useful in motivating medical staff to some extent. Nevertheless, it can also lead to inappropriate behaviors such as induced demand and excessive patient examinations and laboratory testing, resulting in an unreasonable growth of medical expenses, which is not conducive to the improvement of the actual security level of medical insurance [11, 12]. Hence, increasing numbers of areas have begun pilot of a prospective payment system, such as global budget. It has been proven to be more effective in terms of cost reduction than the post payment alternative, [13, 14] because it can stimulate healthcare providers’ cost saving consciousness [15]. (2) Transfer from Fee for Service to Disease Payment, such as Diagnosis Related Groups (DRGs) and quota payments, have proven to be more effective and scientific for standardizing medical expenditures and medical behavior than Fee for Service [16–19].

Gansu province began the payment reform of NRCMS in 2015. The key measures contained two aspects: global budget and quota payments for specific diseases. The principle of global budget is “the total amount is lumpsum and prepaid, while the overpayment is limited”. A quota was paid to medical institutions for the diagnosis, medical examination and treatment services for inpatients suffering from the specific diseases, which were selected according to the disease spectrum and the actual service capability of medical institutions. After the completed therapeutic process, the medical institutions should undertake the excess of quota, and they can reserve the surplus as operating or balancing funds. Medical quality and safety should be ensured according to reasonable standard of clinical pathway. Weiyuan county was among the first batch of pilots in Gansu. First, the health management department determines the total amount of funds available of NRCMS. After deducting the critical illness insurance fund, the risk fund, 15% of the balance in the current year and the total cost of catastrophic diseases, the management department of NRCMS distributes the remaining capital to inpatient and outpatient services, at a proportion of 70 and 30%, respectively. County hospitals and township hospitals provide the inpatient services. The management department determines the yearly total compensation fee for the designated medical institutions according to their average hospitalization expenses, number of inpatients and their medical expenses, growth rate of pooling funds and medical expenses. Then it appropriates 60% of the monthly global budget to institutions as a working capital fund, while pre-paying monthly, settling accounts in the next month and a final account at the end of the year. The total amount of prepayment can be adjusted according to the actual situations. Outpatient services are mainly undertaken by township hospitals and village clinics. The rule of prepayment is similar to hospitalization. Second, the management department brings 170 kinds of hospitalization diseases in county hospitals, 60 in central township hospitals and 50 in general township hospitals into quota payment system. The standard of the quota is calculated according to the cost of each disease in recent 3 years. If the actual cost does not reach the quota standard, the NRCMS pays the quota to the medical institutions, while enrollees pay the out-of-pocket costs at 30% (county level) and 20% (township level) of the actual cost. The balance is paid to the designated medical institutions. However, if the actual cost exceeds the quota standard, the NRCMS will only pay the quota and the designated medical institutions themselves will undertake the excess part while the enrollees pay the out-of-pocket costs at 30% (county level) and 20% (township level) of the quota.

The reform mode of the provider payment system in Weiyuan county is very typical in rural China. As the policy in Weiyuan made more efforts in hospitalization services, our study only focus on the policy effect on inpatients. Yao Jinwen et al. [20] chose Huining county, Gansu, as a sample and evaluated the effect of payment reform and found the hospitalization expense per capita increased 19.15 and 23.36% in county hospitals and township hospitals respectively in 2015 compared to that of 2014. However, during the study period, the policy effect may not have been yet fully manifested, and attention to inpatient’ benefits may have been insufficient. Wang Y X et al [21] chose Jingning county, Gansu as a sample and found that the medical expenses of the inpatients increased 7% per year from 2010 to 2015, and that the average compensation fee increased faster (11% per year) than medical expenses under NRCMS. The proportion of inpatients’ out-of-pocket medical expense decreased from 51.78% (2010) to 39.77% (2015). Because mixed payment system has not been implemented for a long time, the existing empirical studies regarding its effectiveness on medical costs and enrollees’ benefits are neither sufficient nor systematic.

This study evaluates the effectiveness of the “Weiyuan mode”, namely, quota payment for specific diseases under global budget, on the medical cost from the aspects of the total fee, structure of the fee and the enrollees’ benefits. A large database in Weiyuan county was used to examine this important question, while using a relatively innovative method in the area of health services research, which are generalized additive models. Thus, we try to provide suggestions on the practicability of this policy and ways to promote and ameliorate it.

## Methods

### Data source

Weiyuan county was selected as one of the first pilot counties in Gansu province because its economic level represented the average level of the province, and the county had made remarkable achievements in other areas of health reform over the years. Its payment reform mode is one of the key modes of health insurance reform implemented by Gansu province, and definitely typical in rural China. The permanent residential population in Weiyuan was 328.8 thousand in 2016, among which the rural population exceeded 90%. Table 1 shows the basic information about the rural residents’ economic status and health development in the county. The per capita net income of rural residents increased over time atin a relatively high speed. The consumer price index (CPI) in 2016 was improved 0.9% compared to 2014. The service ability of medical institutions was improved, while both the number of annual outpatient visits and inpatient visits increased.

**Table 1:** Economic and health development information of Weiyuan county

	2014	2016
Per capita net income of rural residents (¥)	4535.3	6275.3
Consumer price index (CPI)	102.1	103.0
No. of medical institutions	25	27
No. of hospital beds	1386	1536
No. of health technicians	771	840
No. of annual outpatient visits	485,848	568,536
No. of annual inpatient visits	32,468	47,370

Data for this analysis came from the hospitalization database of NRCMS in Weiyuan County from 2014 to 2016. From this database, information about the inpatients’ medical certificate number, sex, age, admission and discharge date, admission and discharge diagnosis, compensation type and level, total fee and details, out-of-pocket and actual compensation fees could be obtained. A total of 127,491 hospitalization records were included in our analysis. As our data were extracted from the health insurance database, inpatients were not directly involved in this research, and in order to protect their personal privacy, identifiable information was not extracted.

### Study variables

In our study, total fee, actual compensation ratio, out-of-pocket ratio, constituent ratio of the treatment fee, constituent ratio of the inspection and laboratory fee and length of stay were selected as the dependent variables. Since the goal of payment reform is controlling medical costs, [22] the total fee is undoubtedly our main analysis index. Considering the change in price level, the total fee was adjusted through CPI, which can measure the rate at which the prices of consumer goods and services were changing over time, and it is a key statistic for economic and social policymaking [23, 24]. The actual compensation ratio is a key indicator of enrollees’ benefits, as well as the compensation ability of NRCMS and regulation effects for medical institutions, [25, 26] which are deeply affected by medical costs. As the payment to medical institutions for the specific diseases is settled, the out-of-pocket ratio was analyzed as a supplement to assess the effectiveness from the perspective of consumers. In addition, the structure of medical costs was analyzed to evaluate the impact of the reform on doctors’ behavior. In this step, the constituent ratio of the treatment fee and the constituent ratio of the inspection and laboratory fee were chosen as our indexes. Length of stay was selected to reflect service quality and efficiency of medical institutions [27]. Under the premise of ensuring the quality of hospitalization services, effectively shortening the length of stay can make the hospital minimize the cost of resources while reducing the direct and indirect costs of patients, as well as maximizing the comprehensive benefits of the hospital [28–30]. Our independent variable was “year”, which represents the time effect of the reform. Moreover, sex, age, individual attributes and compensation types were introduced as covariates, which were shown to be factors influencing the medical costs [31–34]. Categories of individual attributes are shown in Table 2, which reflect the economic level and payment capacity of enrollees and affect payment rules of NRCMS to medical institutions. Compensation levels vary for the different compensation types (categories shown in Table 2). Among these, only diseases brought into the quota payment system would be afforded by the quota.

**Table 2:** Basic characteristics of inpatients in Weiyuan county, 2014–2016

	2014	2015	2016	*p*-value
***N***	40,568	41,113	45,810	
Sex				
Male	17,010 (41.93%)	17,669 (42.98%)	19,249 (42.02%)	0.008
Female	23,558 (58.07%)	23,444 (57.02%)	26,561 (57.98%)	
Age	43.86 ± 21.31	44.53 ± 21.35	45.01 ± 21.56	< 0.001
Total fee	4008.77 ± 7009.85	4078.50 ± 8419.62	3795.62 ± 7657.61	< 0.001
Length of stay	9.96 ± 9.93	9.68 ± 9.60	9.36 ± 8.08	< 0.001
Actual compensation ratio	58.57 ± 22.39	71.23 ± 42.16	74.48 ± 39.25	< 0.001
Out-of-pocket ratio	36.99 ± 16.02	34.71 ± 16.42	31.37 ± 15.03	< 0.001
Constituent ratio of treatment fee	11.49 ± 17.34	14.44 ± 17.88	19.01 ± 16.68	< 0.001
Constituent ratio of inspection and laboratory fee	11.66 ± 9.76	11.95 ± 9.96	12.39 ± 9.84	< 0.001
Compensation type				
Quota payment diseases	15,679 (38.65%)	26,973 (65.61%)	33,264 (72.61%)	< 0.001
General diseases	20,091 (49.52%)	10,269 (24.98%)	8878 (19.38%)	
Delivery	1940(4.78%)	1158 (2.81%)	1513(3.30%)	
Critical diseases	2691 (6.63%)	1375 (3.34%)	1414 (3.09%)	
Others	167(0.41%)	1338 (3.25%)	741(1.62%)	
Individual attribute				
General family	33,302 (82.09%)	29,601 (72.00%)	31,443 (68.64%)	
Elder people above 60	743(1.83%)	633(1.54%)	711(1.55%)	< 0.001
Low-income Family	2497 (6.16%)	6871 (16.71%)	8952 (19.54%)	
One-child family	1283 (3.16%)	1364 (3.32%)	1525 (3.33%)	
Ligated household with 2 daughters	2189 (5.40%)	2166 (5.27%)	2472 (5.40%)	
Five guarantees family	222 (0.55%)	220 (0.54%)	299 (0.65%)	
The handicapped	330 (0.81%)	243 (0.59%)	224 (0.49%)	
Others	2(0.00%)	15(0.04%)	184(0.40%)	

“Individual attribute” of each enrollee is unique, and the categories were classified by health insurance institutions accurately

### Statistical analysis

Smoothing splines generated in generalized additive models (GAMs) were used to explore the reform effect. As semi-parametric extensions of generalized linear models (GLMs), [35] GAMs use a link function to establish a relationship between the mean of the response variable and a ‘smoothed’ function of the explanatory variables and can deal with non-linear and non-monotonic relationships between the response and the set of explanatory variables [36]. Based on GAMs, we constructed a smooth curve to fit the change of dependent variables under the change of the independent variable (year), as well as introduced covariates one by one to develop the change intuitively [37, 38].

Multiple linear regression models were constructed to estimate the adjusted effects of the reform on medical costs, providers’ behavior and enrollees’ benefits, while conducting stratification analysis for the compounding factors.

Categorical variables are presented as percentages and continuous variables as the means ± SD. Continuous variables were compared using one-way ANOVA, and a Chi-square test was used for categorical variables. The β with a 95% CI is reported as the result of linear regression. All of the analyses were performed with the statistical software packages R (http://www.R-project.org, The R Foundation). A two-sided significance level of 0.05 was used to evaluate the statistical significance.

## Results

### Sample characteristics

Table 2 showed the basic characteristics of rural inpatients of Weiyuan county from 2014 to 2016. The number of female inpatients was higher than the number of male inpatients. The mean age of the inpatients, actual compensation ratio, constituent ratio of the treatment fee, and constituent ratio of the inspection and laboratory fee increased year by year, while length of stay and out-of-pocket ratio decreased. The total fee increased in 2015 but decreased in 2016. Quota payment diseases were settled in 2014, along with another policy called a “tiered medical system” in Weiyuan county; therefore, the “compensation type” defined as “quota payment diseases” was not equal to zero in 2014, and the constituent ratio of it increased along with the official implementation of global budget and quota payment. All differences were significant.

### Smooth curve fitting for the whole sample

**Fig. 1:**
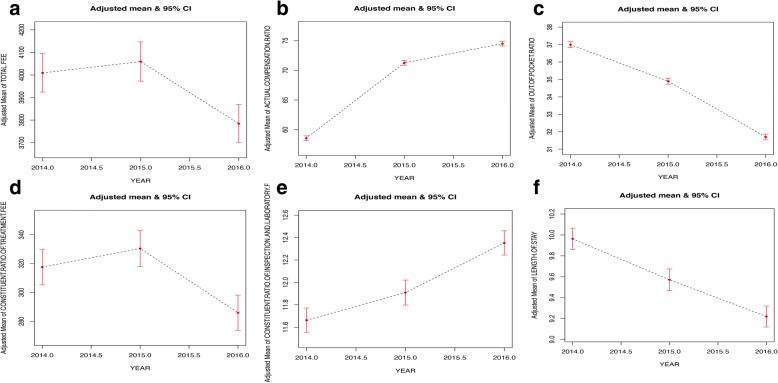
Smooth curve fitting for the whole sample. Ordinate: (**a**) adjusted mean of total fee; (**b**) adjusted mean of actual compensation ratio; (**c**) adjusted mean of out-of-pocket ratio; (**d**) adjusted mean of constituent ratio of treatment fee; (**e**) adjusted mean of constituent ratio of inspection and laboratory fee; (**f**) adjusted mean of length of stay

Figure 1 shows the tendency of the dependent variables by year, with sex, age, individual attribute and compensation type adjusted. Before adjustment, mean of out-of-pocket ratio and length of stay decreased significantly, while the total fee, constituent ratio of the treatment fee, the constituent ratio of the inspection and laboratory fee increased. The mean of the actual compensation ratio increased rapidly in 2015 and decelerated in 2016. After adjusting for the first three variables (sex, age, individual attribute), the curves did not change significantly. When adjusting for the compensation type, the mean of the total fee increased rapidly in 2015, and tended to be stable in 2016. The mean length of stay increased slightly in 2015 and then rapidly decreased in 2016.

### Multiple linear regression models for the whole sample

Table 3 shows the results of multiple linear regression models for the whole sample. Before adjusting, there was a negative correlation between the year and total fee in 2016 (β = − 213.16, 95% CI = − 316.29~ − 110.03) compared with 2014. A negative correlation also existed between the year and out-of-pocket ratio (2015: β = − 2.29, 95% CI = − 2.50~ − 2.07; 2016: β = − 5.62, 95% CI = − 5.84~ − 5.41) as well as length of stay (2015: β = − 0.28, 95% CI = − 0.40~ − 0.15; 2016: β = − 0.60, 95% CI = − 0.72~ − 0.48) both in 2015 and 2016, while absolute value of β in 2016 was larger. There was a positive correlation between the year and actual compensation ratio (2015: β = 12.66, 95% CI = 12.17~ 13.15; 2016: β = 15.91, 95% CI = 15.43~ 16.39), constituent ratio of the treatment fee (2015: β = 2.96, 95% CI = 2.73~ 3.19; 2016: β = 7.53, 95% CI = 7.29~ 7.76) and constituent ratio of the inspection and laboratory fee (2015: β = 0.29, 95% CI = 0.16~ 0.43; 2016: β = 0.73, 95% CI = 0.60~ 0.86), while absolute value of β in 2016 was also bigger. After sex, age and individual attributes were adjusted for, the overall regression result did not change a lot. Nevertheless, after the compensation type was adjusted for, the correlation between year and total fee changed to positive (2015: β = 1122.11, 95% CI = 1018.04~ 1226.17; 2016: β = 1121.19, 95% CI = 1018.37~ 1224.02), while the correlation between year and out-of-pocket ratio changed to not significant in 2015, similar to the length of stay in 2016.

**Table 3:** Results of multiple linear regression models for the whole sample

Crude model		Model I	Model II
Adjusted total fee			
2014	0	0	0
2015	69.72 (−36.14, 175.59)	50.23 (−56.18, 156.64)	1122.11 (1018.04, 1226.17) ***
2016	−213.16 (−316.29, −110.03) ***	−224.61 (−328.94, − 120.29) ***	1121.19 (1018.37, 1224.02) ***
Actual compensation ratio			
2014	0	0	0
2015	12.66 (12.17, 13.15) ***	12.70 (12.2, 13.19) ***	6.04 (5.61, 6.47) ***
2016	15.91 (15.43, 16.39) ***	15.93 (15.44, 16.41) ***	7.13 (6.70, 7.55) ***
Out-of-pocket ratio			
2014	0	0	0
2015	−2.29 (−2.50, −2.07) ***	−2.09 (− 2.31, − 1.88) ***	0.07 (−0.10, 0.25)
2016	−5.62 (−5.84, −5.41) ***	−5.30 (− 5.50, − 5.09) ***	−1.88 (− 2.05, − 1.70) ***
Constituent ratio of treatment fee			
2014	0	0	0
2015	2.96 (2.72, 3.19) ***	3.05 (2.81, 3.28) ***	4.36 (4.13, 4.59) ***
2016	7.53 (7.29, 7.76) ***	7.67 (7.44, 7.90) ***	9.34 (9.12, 9.57) ***
Constituent ratio of inspection and laboratory fee			
2014	0	0	0
2015	0.29 (0.16, 0.43) ***	0.25 (0.11, 0.38) ***	0.53 (0.39, 0.67) ***
2016	0.73 (0.60, 0.86) ***	0.69 (0.56, 0.82) ***	1.14 (1.01, 1.28) ***
Length of stay			
2014	0	0	0
2015	−0.28 (−0.40, −0.15) ***	−0.39 (− 0.52, − 0.27) ***	0.17 (0.04, 0.30) **
2016	− 0.60 (− 0.72, − 0.48) ***	−0.74 (− 0.87, − 0.62) ***	−0.06 (− 0.19, 0.07)

Data in the table: β (95%CI)

***p < 0.01; **0.01 ≤ *p* < 0.05; *p < 0.1

Model I adjusted for sex, age and individual attribute; model II adjusted for compensation type

### Smooth curve fitting for inpatients suffering from quota payment diseases and general diseases

**Fig. 2:**
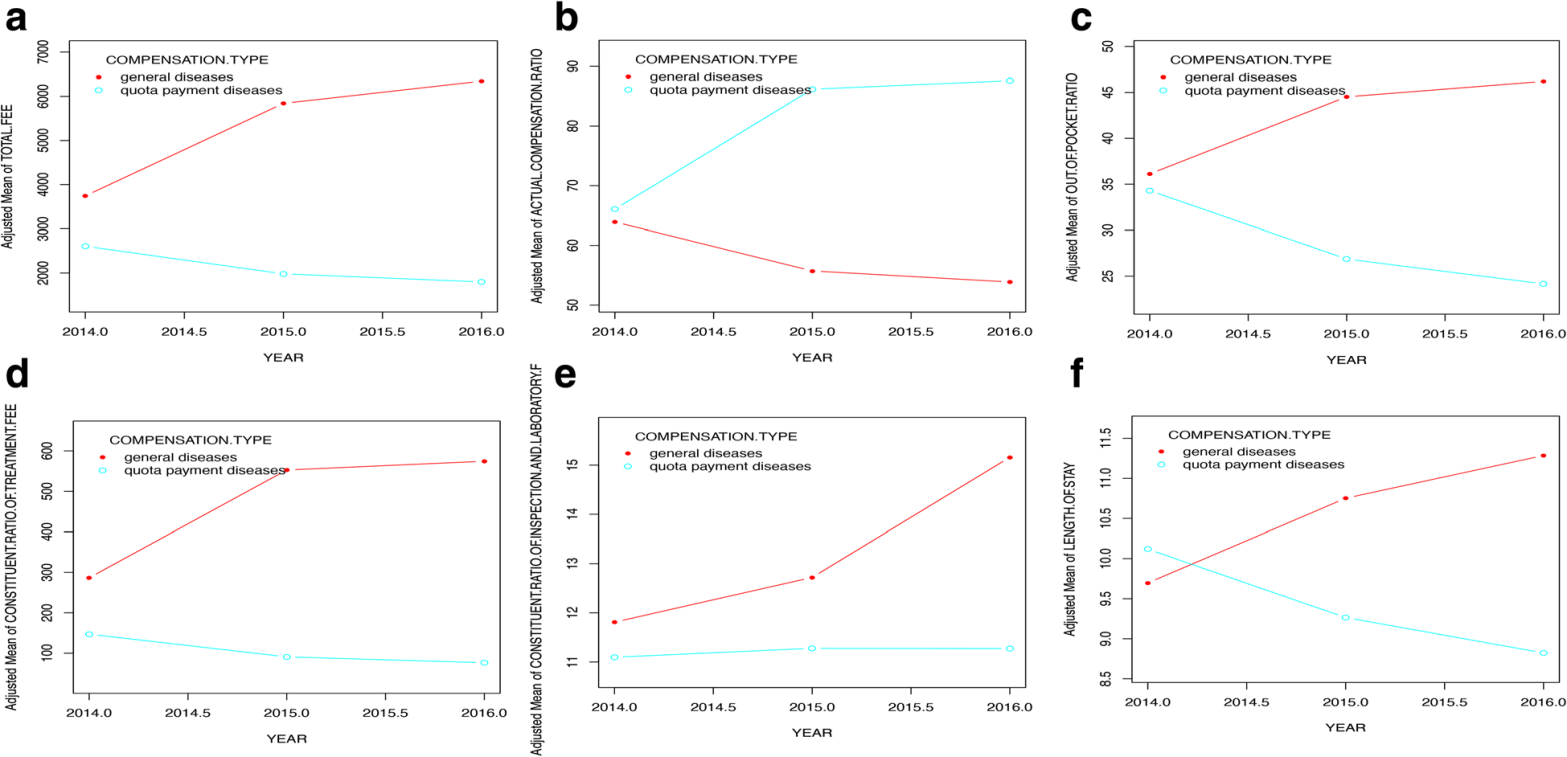
Smooth curve fitting for inpatients suffering from quota payment diseases and general diseases. Ordinate: (**a**) adjusted mean of total fee; (**b**) adjusted mean of actual compensation ratio; (**c**) adjusted mean of out-of-pocket ratio; (**d**) adjusted mean of constituent ratio of treatment fee; (**e**) adjusted mean of constituent ratio of inspection and laboratory fee; (**f**) adjusted mean of length of stay

Considering the objective of this study and the sample size of each category of compensation types, inpatients suffering from quota payment diseases and general diseases were selected as our samples to perform a stratified analysis. “General diseases” were also common diseases, with a lower morbidity compared with “quota payment diseases” and might be brought into quota payment in the future. These two compensation types had similar severity, so we thought they were comparative. Figure 2 shows the significant differences between the two samples after adjusting for sex, age, and individual attributes. For inpatients suffering from quota payment diseases, the actual compensation ratio increased, while it decreased among those suffering from general diseases. The total fee, out-of-pocket ratio and length of stay decreased among quota payment diseases and increased among general diseases. The constituent ratio of the treatment fee increased among both samples. The constituent ratio of the inspection and laboratory fees increased among general diseases and change a little among quota payment diseases.

### Multiple linear regression models for inpatients suffering from quota payment diseases and general diseases

Multiple regression models were repeated for inpatients suffering from quota payment diseases and general diseases. As Table 4 shows, before adjustment, the total fee was negatively correlated with year among inpatients suffering from quota payment diseases (2015: β = − 611.66, 95% CI = − 661.33~ − 561.99; 2016: β = − 810.46, 95% CI = − 858.37~ − 762.55), while positively correlated with year among those suffering from general diseases (2015: β = 2128.88, 95% CI = 1873.82~ 2383.95; 2016: β = 2686.53, 95% CI = 2418.57~ 2954.50). This situation was also reflected in the out-of-pocket ratio (for quota payment diseases, 2015: β = − 7.54, 95% CI = − 7.73~ − 7.36; 2016: β = − 10.35, 95% CI = − 10.53~ − 10.17; for general diseases, 2015: β = 8.26, 95% CI = 7.89~ 8.63; 2016: β = 9.84, 95% CI = 9.45~ 10.23) and length of stay (for quota payment diseases, 2015: β = − 0.77, 95% CI = − 0.91~ − 0.63; 2016: β = − 1.21, 95% CI = − 1.34~ − 1.07; for general diseases, 2015: β = 1.16, 95% CI = 0.93**~** 1.39; 2016: β = 1.80, 95% CI = 1.55~ 2.04). The actual compensation ratio was positively correlated with year among inpatients suffering from quota payment diseases (2015: β = 20.02, 95% CI = 19.3~ 20.74; 2016: β = 21.47, 95% CI = 20.77~ 22.16), while it was negatively associated among the general diseases (2015: β = − 8.29, 95% CI = − 8.66~ − 7.92; 2016: β = − 10.38, 95% CI = − 10.76~ − 9.99). The constituent ratio of the treatment fee and constituent ratio of inspection and laboratory fee were positively correlated with year for both of the samples. After sex, age and individual attributes were adjusted for, the correlation between the year and the constituent ratio of the inspection and laboratory fee in 2016 became not significant among inpatients suffering from quota payment diseases. The other indexes did not change a lot.

**Table 4:** Results of multiple linear regression models for inpatients suffering quota payment diseases and general diseases

Quota payment diseases			General diseases	
Crude model		Adjusted model	Crude model	Adjusted model
Total fee				
2014	0	0	0	0
2015	−611.66 (−661.33, −561.99) ***	− 631.57 (− 681.15, − 581.99) ***	2128.88 (1873.82, 2383.95) ***	2094.25 (1837.18, 2351.33) ***
2016	−810.46 (−858.37, −762.55) ***	− 827.06 (− 875.10, − 779.03) ***	2686.53 (2418.57, 2954.50) ***	2567.26 (2294.38, 2840.14) ***
Actual compensation ratio				
2014	0	0	0	0
2015	20.02 (19.3, 20.74) ***	20.26 (19.55, 20.98) ***	−8.29 (−8.66, −7.92) ***	− 8.57 (− 8.92, − 8.22) ***
2016	21.47 (20.77, 22.16) ***	21.72 (21.03, 22.42) ***	−10.38 (−10.76, −9.99) ***	− 10.85 (− 11.22, − 10.47) ***
Out-of-pocket ratio				
2014	0	0	0	0
2015	−7.54 (− 7.73, − 7.36) ***	−7.47 (− 7.65, − 7.28) ***	8.26 (7.89, 8.63) ***	8.54 (8.19, 8.89) ***
2016	−10.35 (− 10.53, − 10.17) ***	−10.21 (− 10.39, − 10.03) ***	9.84 (9.45, 10.23) ***	10.31 (9.93, 10.68) ***
Constituent ratio of treatment fee				
2014	0	0	0	0
2015	3.48 (3.22, 3.74) ***	3.62 (3.36, 3.87) ***	4.81 (4.42, 5.19) ***	4.90 (4.51, 5.28) ***
2016	9.33 (9.08, 9.58) ***	9.45 (9.2, 9.70) ***	9.74 (9.34, 10.15) ***	10.06 (9.65, 10.47) ***
Constituent ratio of inspection and laboratory fee				
2014	0	0	0	0
2015	0.23 (0.05, 0.41) ***	0.18 (−0.01, 0.36) ***	0.96 (0.71, 1.21) ***	0.93 (0.68, 1.19) ***
2016	0.22 (0.04, 0.40) **	0.17 (−0.01, 0.35) *	3.44 (3.17, 3.70) ***	3.39 (3.12, 3.66) ***
Length of stay				
2014	0	0	0	0
2015	−0.77 (−0.91, − 0.63) ***	−0.81 (− 0.95, − 0.67) ***	1.16 (0.93, 1.39) ***	0.93 (0.69, 1.16) ***
2016	−1.21 (−1.34, −1.07) ***	−1.25 (−1.39, − 1.12) ***	1.80 (1.55, 2.04) ***	1.40 (1.16, 1.65) ***

Data in the table: β (95%CI)

***p < 0.01; **0.01 ≤ *p* < 0.05; *p < 0.1

Adjusted model adjusts for sex, age and individual attribute

## Discussion

In our study, the analysis of GAMs and multiple linear regression showed similar results. Ke H et al.*..* [39] introduced GAMs to fit for hospitalization expenditure and explore influencing factors in 2012. That was the first exploration of GAMs in the area of health services research. Our study further proved that GAMs, which are more widely used in the domains of ecology and epidemiology, [36] are applicable in effect assessment of health policies. Compared to GLMs, GAMs can introduce nonlinear functions, while nonlinearity may make the prediction of independent variables more accurate [40, 41]. In addition, the hypothesis test method of linear models may still be used for GAMs because it is “additive” [40, 42, 43]; therefore, there is a promising application prospect of GAMs in the area of health services research.

Before considering compensation type, our analysis suggests very inspiring results, as both the total fee and out-of-pocket ratio decreased, while the actual compensation ratio increased. Wang P Y et al [44] found the average medical expenses of inpatients in Gansu increased by 23.46% in 2014 compared with 2010 (without taking price increase into consideration), and our study showed an obvious positive effect of the payment reform on containing medical costs. Moreover, the constituent ratio of the treatment fee increased in both samples, which implied doctors’ improving their attention to service quality as well as efficiency; thus, it might have an incentive effect on doctors.

Nevertheless, the results changed when taking compensation type into consideration, mainly reflected in the total fee and length of stay, because the two indexes decreased only among inpatients suffering from quota payment diseases while increased among those with general diseases. Comparing inpatients suffering from quota payment diseases and general diseases, a positive effect mostly reflected among quota payment diseases, because the medical economic burden of this part of inpatients was reduced a lot, while its burden on those suffering from general diseases increased. We can fully acknowledge the effectiveness of the quota payment for specific diseases, but the coverage of these diseases is limited, [45] because it focused on diseases with no complications and relatively separated [46]. As a primitive form of DRGs, quota payment for specific diseases is lacking in scientific assessments of the severity of the diseases, so as to the settlement of the quota. Under quota payment, doctors may take some inappropriate approaches, such as diagnosis upgrades, which means adapting a diagnosis of a disease with a low quota to another disease with a high quota. Decreasing the length of stay is a well-recognized phenomenon of reducing the burden of medical treatment and improving efficiency, and commendably embodies the effect of quota payment for specific diseases in our study, which is consistent with many existing studies [47, 48]. However, we could not ignore the increasing of constituent ratio of inspection and laboratory fees. Although this increase may be caused by doctors’ cautiousness about a diagnosis, there is a great need to evaluate the rationality of examinations and laboratory tests in future research.

Throughout our study, we further proved the importance of a combined provider payment system. Global budget, which has been proven to be an effective cost-control solution around the world, [49] plays an important role in constraining medical costs. However, under the global budget in Weiyuan, NRCMS paid the budget to a single institution, which put forward higher requirements for the regulatory capacity of NRCMS, as well as the stability of inpatient numbers and the disease spectrum [50]. The essence of global budget is to transfer the financial risk from management departments of NRCMS to medical institutions, and thus, medical institutions will have to face the high risks in both financial management and disease treatment [51, 52]. Implementation of quota payment for specific diseases is also essential in Weiyuan’s payment reform. However, as mentioned above, the coverage of disease types is limited. Due to the differences in disease severity, the settlement of the quota needs to be highly accurate, and this is the greatest limitation of this payment method compared to DRGs. At the same time, under the quota payment, doctors’ may pay most of their attention to controlling costs, rather than actively making efforts to improve the health status of residents. In other words, the effectiveness of quota payment is mainly reflected in the process of treatment,rather than in prevention. Thus, quota payment for specific diseases may have deficiencies in the effect of health outcomes, which is precisely the ultimate goal of healthcare reform. We defined the payment reform of quota payment for specific diseases under global budget as the transitional stage, which has already acquired remarkable effects. In the next step of reform, we should make more efforts to evaluate service quality and health outcomes of residents. In addition, we should consider the integration of healthcare systems, including medical institutions (like constructing a medical service community) and services (like integration of disease prevention, treatment, recovery and long-term care) [53–56]. We can use the Kaiser Permanente Medical Care Program in the United States as a reference, [57, 58] to integrate medical institutions,and to pay the budget to the entire medical service community. Thus, medical institutions will not only save costs actively, but also obtain benefits by improving residents’ health status. Based on this, we furtherly implement DRGs to improve the veracity of disease treatment and medical insurance payments. Such a hybrid payment reform may be more sustainable.

### Limitations

This study has several limitations. First, through the inpatient database of NRCMS, the reasonableness of doctors’ behavior cannot be fully assessed, and we cannot make a conclusion as to whether the increased inspection and laboratory fee is appropriate. Second, our data are from 2014 to 2016; however, the policy effect may be delayed, and it may not be totally manifested in our study phase. Third, some confounding factors that influence the reform effect may not be included in the database. Fourth, this paper only assessed the effect of payment reform within county in China. Finally, we could not assess the reform impact on residents’ actual health status in our study.

## Conclusions

GAMs are relatively new method to assess policy effects in the area of health services research, but they are practical and rigorous with bringing covariates into model one by one and can exhibit the time effect of policies intuitively by a smooth fitting curve. Additionally, the combination of global budget and quota payment for specific diseases has achieved obvious effects on cost control in Weiyuan, Gansu. We should evaluate the long-term effect of this reform pattern and its replicability, as well as adapt it to the objective of improving residents’ health status.

## Abbreviations

DRGs: Diagnosis Related Groups
GAMs: generalized additive models
NRCMS: new rural cooperative medical system
